# 5-HT_2C_Rs Expressed by Pro-Opiomelanocortin Neurons Regulate Energy Homeostasis

**DOI:** 10.1016/j.neuron.2008.09.033

**Published:** 2008-11-26

**Authors:** Yong Xu, Juli E. Jones, Daisuke Kohno, Kevin W. Williams, Charlotte E. Lee, Michelle J. Choi, Jason G. Anderson, Lora K. Heisler, Jeffrey M. Zigman, Bradford B. Lowell, Joel K. Elmquist

**Affiliations:** 1 Division of Hypothalamic Research, Departments of Internal Medicine, Pharmacology, and Psychiatry, The University of Texas Southwestern Medical Center, Dallas, TX 75390, USA; 2 Department of Medicine, Beth Israel Deaconess Medical Center, Harvard Medical School, Boston, MA 02215, USA; 3 Department of Pharmacology, University of Cambridge, Cambridge CB2 1PD, UK

**Keywords:** HUMDISEASE, MOLNEURO, SIGNALING

## Abstract

Drugs activating 5-hydroxytryptamine 2C receptors (5-HT_2C_Rs) potently suppress appetite, but the underlying mechanisms for these effects are not fully understood. To tackle this issue, we generated mice with global *5-HT_2C_R* deficiency (*2C* null) and mice with 5-HT_2C_Rs re-expression only in pro-opiomelanocortin (POMC) neurons (*2C/POMC* mice). We show that *2C* null mice predictably developed hyperphagia, hyperactivity, and obesity and showed attenuated responses to anorexigenic 5-HT drugs. Remarkably, all these deficiencies were normalized in *2C/POMC* mice. These results demonstrate that 5-HT_2C_R expression solely in POMC neurons is sufficient to mediate effects of serotoninergic compounds on food intake. The findings also highlight the physiological relevance of the 5-HT_2C_R-melanocortin circuitry in the long-term regulation of energy balance.

## Introduction

The central 5-hydroxytryptamine (5-HT) system, including the 5-HT_2C_ receptors (5-HT_2C_Rs) plays critical roles in the regulation of energy homeostasis. This is best demonstrated by the hyperphagia and obesity in mice with global *5-HT_2C_R* deficiency (Nonogaki et al., 1998; Tecott et al., 1995). 5-HT_2C_Rs also contribute to the anorexigenic effects of d-fenfluramine (d-Fen) (Vickers et al., 1999), a drug that was widely prescribed and was clinically effective to combat obesity in the 1990s. However, the drug was withdrawn from clinical use due to adverse cardiopulmonary events (Connolly et al., 1997). Nonetheless, the efficacy of this drug regimen in humans underscores the importance of the central 5-HT system in regulating energy balance and the need to understand the mechanisms underlying its effects. More recently, commonly used atypical antipsychotic drugs (AAPDs) have been reported to cause serious weight gain, which may be associated with their 5-HT_2C_R antagonist properties and with polymorphisms in the *5-HT_2C_R* gene (Reynolds et al., 2002; Templeman et al., 2005). Furthermore, a splicing variant of 5-HT_2C_Rs with impaired function has been suggested to contribute to hyperphagia and obesity in patients with Prader-Willi syndrome (Kishore and Stamm, 2006). Collectively, these observations strongly suggest that an improved understanding of the mechanisms by which 5-HT_2C_Rs regulate feeding behavior and body weight homeostasis may not only lead to the development of antiobesity drugs with fewer side effects but also may facilitate countering the metabolic deficits commonly seen in AAPD consumers or patients with Prader-Willi syndrome.

Pro-opiomelanocortin (POMC) neurons in the arcuate nucleus of hypothalamus (ARC) produce α-melanocyte-stimulating hormone (α-MSH), an endogenous agonist of melanocortin 4 receptors (MC4Rs) (Cone, 2005; Elmquist et al., 1999; Williams and Schwartz, 2005). The central melanocortin system is required to maintain food intake, body weight, and glucose homeostasis (Cone, 2005; Elmquist et al., 1999; Williams and Schwartz, 2005; Yeo et al., 2000).

The melanocortin pathway has been hypothesized to be downstream of 5-HT_2C_Rs and mediate the effects of 5-HT_2C_Rs on feeding behavior. Particularly, POMC neurons in the ARC coexpress 5-HT_2C_Rs (Heisler et al., 2002) and receive inputs from 5-HT-immunoreactive nerve terminals (Kiss et al., 1984). This anatomical evidence indicates that central 5-HT is positioned to directly act on POMC neurons via 5-HT_2C_Rs. The potential role of 5-HT_2C_Rs in POMC neurons is supported by electrophysiological studies showing that 5-HT drugs, including d-Fen, activate POMC neurons via 5-HT_2C_R-mediated mechanisms (Heisler et al., 2002; Qiu et al., 2007). In addition, 5-HT_2C_R agonists stimulate POMC expression in the ARC (Lam et al., 2008; Zhou et al., 2007). Finally, we have previously shown that the anorexigenic effect of d-Fen is blunted in mice lacking MC4Rs (Heisler et al., 2006). Collectively, these findings suggest that the intact central melanocortin pathway is required for the acute actions of 5-HT_2C_Rs to regulate energy balance. However, whether direct 5-HT action only on POMC neurons is sufficient to mediate potent anorexigenic effects of 5-HT compounds is unknown. In addition, the physiological significance of the interaction of the central melanocortin system and 5-HT_2C_Rs in regulating long-term energy balance remains to be established.

To directly address these questions, we generated mice with global deficiency of *5-HT_2C_R* (*2C* null) and mice with 5-HT_2C_Rs re-expressed specifically and only in POMC neurons (*2C/POMC*). Taking advantage of these unique genetic models, we directly tested the hypotheses that 5-HT_2C_Rs in POMC neurons mediate the anorexigenic effects of 5-HT agents and that the re-establishment of the 5-HT_2C_R-melanocortin circuitry is sufficient to rescue the obese phenotypes caused by global *5-HT_2C_R* deficiency.

## Results and Discussion

**Figure 1:**
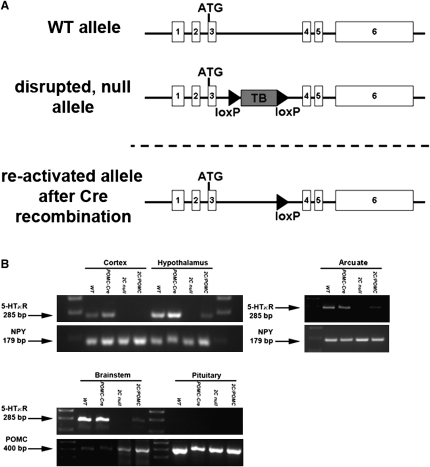
Generation of *loxTB 5-HT_2C_R* Mice (A) A disrupted *5-HT_2C_R* allele was generated by inserting a *loxP*-flanked transcriptional blocker (*loxTB*) between exons 3 and exon 4 of the *5-HT_2C_R* gene. Expression of *Cre*-recombinase removes the transcriptional blocker and allows *5-HT_2C_R* expression. (B) Messenger RNAs of *5-HT_2C_R* and *neuropeptide Y* (*NPY*) or *POMC* were detected with RT-PCR in the cerebral cortex, whole hypothalamus, ARC, brainstem, and pituitary of WT, *POMC-Cre*, *2C* null, and *2C/POMC* mice.

*2C* null (*loxTB 5-HT_2C_R*) mice were generated by inserting a *loxP*-flanked transcription blocker (*loxTB*) (Balthasar et al., 2005; Zigman et al., 2005) into the *5-HT_2C_R* gene to globally disrupt its expression. Crossing *2C* null mice with *POMC-Cre* mice (Balthasar et al., 2004) produced *2C/POMC* mice, in which expression of endogenous 5-HT_2C_Rs was reactivated selectively in POMC neurons by *Cre*-recombinase (Figure 1A).

Using PCR primers specific for *5-HT_2C_R* mRNA, we found that expression of *5-HT_2C_R* mRNA was disrupted in the cerebral cortex, whole hypothalamus, ARC, and brainstem of *2C* null mice. In *2C/POMC* mice, *5-HT_2C_R* mRNA was re-expressed only in samples of the whole hypothalamus or brainstem. Further, we found specific re-expression in samples of microdissected arcuate nucleus, where POMC neurons are located (Bronstein et al., 1992; Elias et al., 1999). We found no re-expression in the cerebral cortex (Figure 1B). Thus, *5-HT_2C_R* mRNA was re-expressed in a POMC-specific manner. Although *POMC* mRNA is expressed in the anterior pituitary gland, we found no endogenous *5-HT_2C_R* mRNA expressed in the pituitary of wild-type (WT) mice. It should be noted that *POMC* is also expressed by a small population of neurons in the nucleus of solitary tract (NTS) in the brainstem (Fan et al., 2004; Huo et al., 2006), a brain region regulating feeding and satiety. Thus, in addition to 5-HT_2C_Rs expressed by ARC POMC neurons, we cannot rule out that the small number of POMC neurons in the NTS may contribute to the responses outlined below. We were not able to histologically validate re-expression of 5-HT_2C_Rs specifically in POMC neurons due to the lack of highly selective reagents such as 5-HT_2C_R antibodies or ligands specific for 5-HT_2C_R binding.

**Figure 2:**
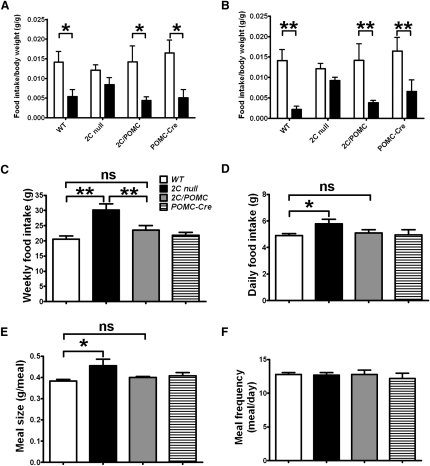
5-HT_2C_Rs in POMC Neurons Rescue the Attenuated Anorexigenic Effects of 5-HT Agents, Hyperphagia and Decreased Satiety in *2C* Null Mice Four-month-old body-weight-matched HFD-fed mice (n = 6–9 per genotype) were fasted overnight, and then saline, d-Fen (3 mg/kg) (A), or mCPP (5 mg/kg) (B) were intraperitoneally injected 30 min prior to presentation of HFD. Food intake in the following hour was measured and normalized by their body weight. (C) Weekly food intake was measured in singly housed HFD-fed mice (n = 5–7 per genotype) at 11 weeks. *2C* null mice consumed significantly more diet than WT, *2C/POMC*, and *POMC-Cre* mice, and there was no significant difference in food intake of WT, *2C/POMC*, and *POMC-Cre* mice. Daily food intake (D), meal size (E), and meal frequency (F) of 3-month-old chow-fed mice (n = 5–11 per genotype) were measured with the TSE system. *2C* null mice had significantly increased food intake and meal size than WT, and these parameters were not significantly different among WT, *2C/POMC*, and *POMC-Cre* mice. There was no significant difference in meal frequency among the four genotypes. Data are presented as mean ± SEM, and ^∗^p < 0.05 and ^∗∗^p < 0.01 in one-way ANOVA analysis with Student-Newman-Keuls post hoc comparison.

To further confirm that our *2C* null mice lack 5-HT_2C_Rs, we assessed the acute effects on food intake to anorexigenic 5-HT agents in *2C* null and WT mice. We found that while d-Fen significantly reduced 1 hr food intake in WT mice, this acute anorexigenic effect was attenuated in *2C* null mice (Figure 2A). Similarly, an agonist of 5-HT_2C_Rs and 5-HT 1B receptors, meta-chlorophenylpiperazine (mCPP) (Hewitt et al., 2002), decreased food intake in WT mice, an effect that was blunted in *2C* null mice (Figure 2B). These findings are consistent with the results obtained in conventional *5-HT_2C_R* knockout mice that showed attenuated anorexigenic effects in response to both d-Fen and mCPP (Tecott et al., 1995; Vickers et al., 1999).

To determine whether selective re-expression of 5-HT_2C_Rs in POMC neurons is sufficient to restore the anorexigenic responses to 5-HT drugs, we assessed *2C/POMC* mice, whose 5-HT_2C_Rs were re-expressed selectively in POMC neurons. Notably, the anorexigenic effects induced by both d-Fen and mCPP were restored to levels that were indistinguishable from those in WT mice (Figures 2A and 2B). Thus, in line with our previous finding that the anorexigenic effect of d-Fen is blunted in mice lacking MC4Rs (Heisler et al., 2006), the current results indicate that 5-HT_2C_Rs expressed by POMC neurons are sufficient to mediate the acute anorexigenic effects of 5-HT compounds.

5-HT_2C_Rs are also physiological regulators of feeding, as *5-HT_2C_R* knockout mice are hyperphagic (Nonogaki et al., 1998; Tecott et al., 1995). Consistent with these observations, our *2C* null mice showed a hyperphagic phenotype. Specifically, a 50% increase in weekly food intake was observed in high fat diet (HFD)-fed *2C* null mice compared to their WT littermates at 11 weeks (Figures 2C). Similarly, 12-week-old chow-fed *2C* null mice were hyperphagic (Figure 2D). Remarkably, food intake of *2C/POMC* mice on HFD or chow was comparable to that of WT mice (Figures 2C and 2D). These findings demonstrate that 5-HT_2C_Rs expressed by POMC neurons are sufficient to normalize the hyperphagia characteristic of mice with *5-HT_2C_R* deficiency.

Previously, it has been reported that mCPP-induced suppression in food intake in mice is associated with an early termination of feeding behavior, an effect that was partially reversed by a selective 5-HT_2C_R antagonist (Hewitt et al., 2002). Similarly, d-Fen produces an advanced satiation in WT mice, and this response is attenuated in mice with *5-HT_2C_R* deficiency (Vickers et al., 1999). These findings suggest that activation of 5-HT_2C_Rs enhances satiety signals to terminate food intake. Consistent with this notion, here we show that meal size of *2C* null mice was significantly bigger than that of WT littermates, while their meal frequency was comparable to that of WT mice (Figures 2E and 2F). However, meal size of *2C/POMC* mice was not statistically different from that of WT mice, which indicates that 5-HT_2C_Rs re-expressed in POMC neurons are sufficient to advance the satiation process and therefore suppress feeding. Interestingly, a proportion of POMC neurons in the ARC have been shown to project to the NTS in the brainstem to potentiate satiety signals (Zheng et al., 2005). Thus, it is plausible that endogenous 5-HT, by acting on 5-HT_2C_Rs in the ARC POMC neurons, activates POMC neurons innervating the NTS to increase satiety and suppress food intake. However, another possibility that we cannot exclude is that the 5-HT_2C_R-induced satiation is, at least partly, mediated by POMC neurons in the NTS, since 5-HT_2C_Rs are also reactivated in NTS POMC neurons in *2C/POMC* mice.

**Figure 3:**
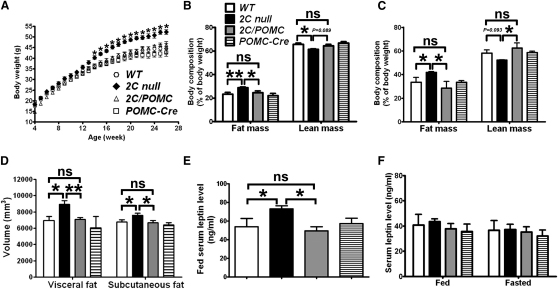
5-HT_2C_Rs in POMC Neurons Rescue Obesity, Hyperadiposity, and Hyperleptinemia in *2C* Null Mice (A) Weekly body weight was measured in group-housed mice fed with HFD (n = 20–39 per genotype). Body weight of *2C* null mice started to be significantly higher than that of WT, *2C/POMC*, and *POMC-Cre* mice from 14 weeks of age, and there was no significant difference in body weight of WT, *2C/POMC*, and *POMC-Cre* mice. *2C* null mice fed with HFD accumulated significantly higher fat mass than WT, *2C/POMC*, and *POMC-Cre* littermates at week 13 (B) and at week 28 (C), and lean mass was significantly decreased in young *2C* null mice and tended to decrease in old *2C* null mice; there was no significant difference in fat mass and lean mass among WT, *2C/POMC*, and *POMC-Cre* mice. N = 6–10 per genotype in (B) and (C). (D) 23-week-old HFD-fed *2C* null mice showed significantly increased visceral and subcutaneous fat storage than WT, *2C/POMC*, and *POMC-Cre* mice, and there was no significant difference in fat distribution in WT, *2C/POMC*, and *POMC-Cre* mice. N = 4–5 per genotype in (D). (E) Eight-month-old HFD-fed *2C* null mice had significantly higher leptin level than WT, *2C/POMC*, and *POMC-Cre* mice, and there was no significant difference in leptin levels of WT, *2C/POMC*, and *POMC-Cre* mice. N = 4–5 per genotype in (E). (F) There is no significant difference in fed and fasted serum leptin levels in 4-month-old HFD-fed mice (n = 5–6 per genotype). Data are presented as mean ± SEM, and ^∗^p < 0.05 and ^∗∗^p < 0.01 in one-way ANOVA analysis with Student-Newman-Keuls post hoc comparison.

To test the significance of the 5-HT_2C_R-melanocortin circuitry in the long-term regulation of energy homeostasis, we measured the body weight and body composition of WT, *2C* null, and *2C/POMC* mice. Global deletion of 5-HT_2C_Rs leads to late-onset obesity in chow-fed mice, a phenotype that can be accelerated by HFD-feeding (Nonogaki et al., 1998). In agreement, we found that our *2C* null mice fed with HFD showed significant differences in body weight compared to their WT littermates starting at 14 weeks of age, and by week 26, *2C* null mice weighed 8 g more than WT mice (Figure 3A). Deletion of 5-HT_2C_Rs also results in alterations in adiposity. We found a significant increase in fat mass in *2C* null mice as early as 13 weeks of age, which was accompanied by a decrease in lean mass (Figure 3B). At week 28, *2C* null mice accumulated significantly more body fat mass than WT mice, and their body lean mass tended to decrease (Figure 3C). We used CT scans to further confirm that *2C* null mice had significantly increased adiposity in both visceral and subcutaneous depots (Figure 3D). Notably, we found that selective re-expression of 5-HT_2C_Rs in POMC neurons was sufficient to rescue the obese phenotype observed in *2C* null mice. Particularly, HFD-fed *2C/POMC* mice had comparable body weight as WT mice (Figure 3A). Both young and old *2C/POMC* mice had similar levels of adiposity as age-matched WT littermates (Figures 3B and 3C). In addition, visceral and subcutaneous fat distributions in old *2C/POMC* mice were similar to WT mice (Figure 3D). Thus, these findings demonstrate that 5-HT_2C_Rs re-expressed in POMC neurons are sufficient to rescue obesity caused by global *5-HT_2C_R* deficiency.

Secondary to obesity and increased fat mass, mice lacking 5-HT_2C_Rs have been shown to develop late-onset hyperleptinemia and leptin insensitivity (Nonogaki et al., 1998). Consistently, we detected significantly increased serum leptin in old HFD-fed *2C* null mice (Figure 3E). Young *2C* null mice had similar serum leptin levels as WT littermates (Figure 3F). Re-expression of 5-HT_2C_Rs in POMC neurons normalized serum leptin level in old HFD-fed *2C/POMC* mice (Figure 3E).

**Figure 4:**
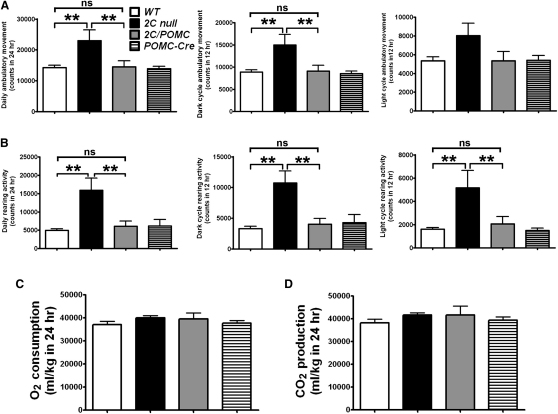
5-HT_2C_Rs in POMC Neurons Rescue Hyperactivity in *2C* Null Mice (A) Three-month-old chow-fed *2C* null mice showed significantly more ambulatory movement than WT, *2C/POMC*, and *POMC-Cre* mice over the entire 24 hr (left panel) and 12 hr dark cycle (middle panel), but no significant difference was observed over the 12 hr light cycle (right panel). (B) *2C* null mice showed significantly more rearing activity than WT, *2C/POMC*, and *POMC-Cre* mice over the entire 24 hr (left panel), 12 hr dark cycle (middle panel), and 12 hr light cycle (right panel). There was no significant difference in ambulatory movement and rearing activity of WT, *2C/POMC*, and *POMC-Cre* mice. O_2_ consumption (C) and CO_2_ production (D) were not significantly different among four genotypes. N = 5–11 per genotype. Data are presented as mean ± SEM, and ^∗∗^p < 0.01 in one-way ANOVA analysis with Student-Newman-Keuls post hoc comparison.

It has been reported that young pre-obese *5-HT_2C_R* knockout mice also display elevated physical activity (Nonogaki et al., 2003). However, young mutant mice display normal energy expenditure (Nonogaki et al., 2003). Consistent with these observations, we found that ambulatory movements and rearing activities of young chow-fed *2C* null mice were significantly increased compared to WT littermates (Figures 4A and 4B). We found no difference in the energy expenditure as measured by O_2_ consumption and CO_2_ production (Figures 4C and 4D). Notably, the hyperactivity of *2C* null mice was completely normalized in *2C/POMC* mice (Figures 4A and 4B). These data indicate that selective re-expression of 5-HT_2C_Rs in POMC neurons is sufficient to normalize the hyperactivity in mice lacking 5-HT_2C_Rs.

In addition to hyperphagia and obesity, another characteristic phenotype of mice with *5-HT_2C_R* deficiency is spontaneous seizures, which result in a high mortality rate in relatively young mice (Tecott et al., 1995). Although we did not quantitatively analyze the epileptic phenotype in our mice, we anecdotally observed several episodes of tonic-clonic seizures in both *2C* null and *2C/POMC* mice. In addition, similar to *5-HT_2C_R* knockout mice (Tecott et al., 1995), our *2C* null mice displayed a significant decreased survival rate compared to WT mice (see the Supplemental Data available online). Interestingly, the survival rate of HFD-fed *2C* null mice is significantly higher than that of *2C* null mice on chow (see Supplemental Data). This is interesting given the fact that epileptic patients can benefit from ketogenic diets (Kim do and Rho, 2008). Notably, *2C/POMC* mice showed a similar survival rate to their *2C* null littermates, regardless whether they were fed with HFD or chow (see Supplemental Data). Therefore, these results suggest that re-expression of 5-HT_2C_Rs in POMC neurons is not sufficient to rescue the epileptic phenotype characteristic of *5-HT_2C_R* knockout mice. One may argue that the seizures and the associated high mortality rate may confound the interpretation of the metabolic measurements in *2C* null and *2C/POMC* mice. However, given the likely metabolic expense of seizures, we would suggest that the seizure phenotype would mask the observed obese phenotype in *5-HT_2C_R* knockout mice. Finally, it is important to note that *2C* null and *2C/POMC* mice displayed distinct metabolic profiles despite the fact that they suffered almost identical mortality rate (presumably due to similar epileptic status).

Serotoninergic neurons have broad projections that innervate the entire central nervous system and regulate diverse behaviors including feeding. For years, research focused on the mechanisms of the central 5-HT system, including 5-HT_2C_Rs, has been one of the priorities in the obesity field because of the potent effects of serotoninergic agents on feeding. Indeed, drugs generally influencing 5-HT bioavailability, such as sibutramine, are among the few approved current pharmacotherapies for obesity. However, due to the widespread distribution of 5-HT_2C_Rs in the brain (Molineaux et al., 1989) and the lack of commercially available 5-HT_2C_R-selective drugs, discerning mechanisms underlying functions of 5-HT_2C_Rs has proven particularly difficult. Here, taking advantage of unique genetic mouse models, we have provided direct evidence that POMC neurons are physiologically important targets of potent anorexigenic 5-HT compounds such as d-Fen to suppress food intake. In addition, our results highlight the importance of the central 5-HT_2C_Rs expressed by POMC neurons in maintaining normal feeding behavior and body weight homeostasis. However, our findings do not demonstrate that POMC neurons are the only site where 5-HT_2C_Rs regulate energy balance, as our results do not exclude the possibility that other redundant neuronal populations expressing 5-HT_2C_Rs may also be sufficient to mediate similar effects. For example, in addition to the ARC, 5-HT_2C_Rs are also found in other brain regions implicated in the regulation of body weight homeostasis, including the paraventricular nucleus, ventromedial nucleus, dorsomedial nucleus, lateral hypothalamic area, and parabrachial nucleus (Hoffman and Mezey, 1989; Pasqualetti et al., 1999; Wright et al., 1995). These regions all receive serotoninergic projections (Petrov et al., 1992; Steinbusch and Nieuwenhuys, 1981). The physiological relevance of 5-HT_2C_R expression in these sites is yet to be characterized. However, our unique mouse model will allow us to directly address the importance of 5-HT_2C_Rs in any site in the central nervous system in which specific expression of *Cre*-recombinase can be directed.

## Experimental Procedures

### Generation of *2C* Null and *2C/POMC* Mice

*2C* null (*LoxTB 5-HT_2C_R*) mice were created by inserting a *loxP*-flanked transcriptional blocking cassette (*loxTB*) (Balthasar et al., 2005; Zigman et al., 2005) between the exons 3 and 4 of the *X*-linked *5-HT_2C_R* gene (Figure 1A). The targeting construct was generated using ET cloning and related technologies within EL250 cells and the BAC that contains the *5-HT_2C_R* gene (Mouse RPCI.22 BAC clone 32 B 10) (Invitrogen, Carlsbad, CA). The *loxTB 5-HT_2C_R* gene was placed in a pGEM-T plasmid using the following PCR template (left homology arm [TAATTATAAACACTATTATACACAGAGATTTCCAATTTATTAACTAAAATTACTTTCAAAGTCATGCCTTACCGG TCCAACGCGTTGGATGCATAG] and right homology arm [GAGCTTAAAACATTAGCAATCAGCAGCAAAGATGCA AATATTCCTCAACCGTGTCAGTACTATAGACTAAACCGGTGTATTTTCTCCTTACGCATC]) and then linearized. The final targeting construct, which consisted of the *5-HT_2C_R-loxTB* flanked by 4 kb *5-HT_2C_R* homology arms, was electroporated into ES cells, and correct targeting was confirmed by Southern blot analyses. For Southern blot analysis, ES cells were digested with NCO1 for 4 hr at 37°C. Primers were labeled with Megaprime DNA labeling kit. The right probe was 202 bp and amplified using TCACAATTGAAGACATTTCCTG and TGTTGGGTTTCTTTGTGGTTCTC. Detection of a nontargeted ES cell would produce an 11,095 bp band, and a target ES cell would produce a 6718 bp band. The left probe was 265 bp and amplified using TGTGAGAAATGCTGCAGGAATAAA and TTGGGAAGTTTTGTTTTTGTGGA. Detection of a nontargeted ES cell would produce an 11,095 bp band, while a targeted ES cell would produce a 6570 bp band. Targeted ES cells were injected into blastocysts, and, after germline transmission was established, the chimera carrying the recombinant allele was crossed onto a C57BL/6J background.

Female *loxTB 5-HT_2C_R* heterozygous mice (backcrossed to the C57BL/6J background for eight generations) were crossed with male *POMC-Cre* hemizygous mice (backcrossed to the C57BL/6J background for five generations), and only male offspring were collected for the experiments described below. These male mice carry one of the following genotypes: wild-type (WT), hemizygous *loxTB 5-HT_2C_R* (*2C* null), hemizygous *loxTB 5-HT_2C_R* and hemizygous *POMC-Cre* (*2C/POMC*), as well as hemizygous *POMC-Cre* (*POMC-Cre*). All mice were housed in their home cages with food and water available ad libitum in a temperature-controlled room with 12 hr light, 12 hr dark cycle in the animal facility of University of Texas Southwestern Medical Center at Dallas.

### Food Intake and Body Weight

For the HFD-feeding study, mice were group housed (two to five mice per cage) and provided with a 42% fat diet (TD.88137, Harlan Teklad) from 5 weeks of age, and body weight was measured weekly. Another HFD cohort was individually housed, and a similar growth pattern was observed as in the group-housed cohort. Food intake was measured weekly from mice that were individually housed.

### RT-PCR

The cerebral cortex, whole hypothalamus, ARC, brainstem, and pituitary were taken from WT, *2C* null, *2C/POMC*, and *POMC-Cre* mice. Total RNA was extracted with the QIAzol Lysis Reagent (QIAGEN, Valencia, CA) and reverse-transcribed to cDNA using SuperScript II First-Strand cDNA Synthesis kit (Invitrogen, Carlsbad, CA) according to the manufacturer's instructions. Expression of *5-HT_2C_R* was detected in all the tissues with primers that span exon 3 and 4 of *5-HT_2C_R* gene (forward, CTCACTCCTTGTGCACCT; reverse, CCCACCAGCATATCAGCAATG). As positive controls, expression of NPY was detected in the cerebral cortex, whole hypothalamus, and ARC with NPY primers (forward, TGCTCGTGTGTTTGGGCATTCTGG; reverse, GAGACACTGATTTCAGACCTC); POMC was detected in the brainstem and pituitary using POMC primers (forward, CAGACCTCCATAGATGTGTGGAGC; reverse, CTCAGCAACGTTGGGGTACAC). Due to the low level of *5-HT_2C_R* mRNA in the ARC of *2C/POMC* mice, the first PCR products of ARC tissues from all mice were purified and subjected to the second PCR amplification using the same primers. *5-HT_2C_R* PCR amplicons from the hypothalami of WT, *2C/POMC*, and *POMC-Cre* mice were gel purified and sequenced in the local core facility, and we confirmed that *5-HT_2C_R* mRNA re-expressed in *2C/POMC* hypothalamus was identical to those of WT and *POMC-Cre* hypothalami.

### Body Composition and Fat Distribution

Body composition was measured with the Bruker minispec mq10 MRS system. For fat distribution, mice were anesthetized with 1% isoflurane inhalation and then the trunk (from base of the skull as the spinal canal begins to widen and the distal end of the tibia) of each mouse was scanned at an isotropic voxel size of 93 μm (80 kV, 450 μA, and 100 ms integration time) using the eXplore Locus micro-CT scanner (GE Health Care). Three-dimensional images were reconstructed from two-dimensional grayscale image slices and visualized using Microview Software (GE Medical System). Density values for soft tissue and bone were calibrated from a phantom (GE Health Care) containing air bubble, water, and hydroxyl apatite rod. The separation of fat regions was obtained from the appropriate grayscale value (upper threshold, −165; lower threshold, −360). The abdominal muscular wall was used as the differentiation line to separate visceral adipose tissue from subcutaneous adipose tissue. The contour lines were drawn around the viscera and three-dimensional ROI was generated. The visceral fat was determined from the histogram of these segmented viscera using the same thresholds. Subcutaneous fat was obtained by subtracting visceral fat from the total body fat.

### Acute Anorexigenic Responses to 5-HT Compounds

To assess whether 5-HT_2C_Rs in POMC neurons are sufficient to mediate anorexigenic effects of 5-HT compounds, 4-month-old HFD-fed mice were individually housed and weight matched. After overnight fasting, the mice received intraperitoneal injections of saline, d-Fen (3 mg/kg), or mCPP (5 mg/kg). HFD was represented to their cages 30 min after the injections. Food intake in the next hour was measured and normalized by their body weight. Each mouse was tested with all three treatments (d-Fen, mCPP, and saline), administered in a counterbalanced order, with a minimum of 5 days between the treatments. Data were presented as mean ± SEM. Differences among genotypes were determined by two-way ANOVA analysis, followed by the post hoc Student-Newman-Keuls test.

### Serum Leptin

Fed and/or fasted serum leptin levels were measured in HFD-fed mice at 4 or 8 months of age. At month 4, food was removed from the home cages for 2 hr (fed condition) or for overnight (fasted condition), and blood was collected from the saphenous vein. At month 8, trunk blood was collected 2 hr after mice were deprived of food. Leptin levels were measured using the leptin ELISA kit (Crystal Chem Inc., Downers Grove, IL) according to the manufacturer's instruction.

### Physical Activity, Energy Expenditure, and Meal Patterns

Physical activity, energy expenditure, and meal patterns were monitored using a combined indirect calorimetry system (TSE Systems GmbH, Bad Homburg, Germany) (Pfluger et al., 2008). After adaptation for 6 days, physical activity was determined for 4 days using a multidimensional infrared light beam system with beams installed on cage bottom and cage top levels. Ambulatory movement was defined as breaks of any two different light beams at cage bottom level, while rearing was recorded once the mouse broke any light beam at the top level. Simultaneously, O_2_ consumption and CO_2_ production were measured to determine the energy expenditure. In addition, meal patterns were determined continuously by integration of weighing sensors fixed at the top of the cage from which the food containers have been suspended into the sealed cage environment. Meals were defined as food intake events with a minimum duration of 60 s and a break of 300 s between food intake events.

### Statistical Analysis

Data were presented as mean ± SEM. Statistical analyses were carried out with SigmaStat software. Unless otherwise mentioned, all data were analyzed by one-way ANOVA analysis, followed by the post hoc Student-Newman-Keuls test when the ANOVA analysis indicated significant differences. p < 0.05 indicated statistical significance.
