# Supplementary material for: 5-HT2CRs Expressed by Pro-Opiomelanocortin Neurons Regulate Energy Homeostasis
Source: Neuron. 2008 Nov 26;60(4-2):582–9. doi: 10.1016/j.neuron.2008.09.033 (PMC2631191; doi:10.1016/j.neuron.2008.09.033)
Supplement: Document S1. Supplemental Figure [file mmc1.pdf]

Neuron, Volume *60*

Supplemental Data

5-HT$_{2C}$Rs Expressed by Pro-Opiomelanocortin Neurons Regulate Energy Homeostasis

Yong Xu, Juli E. Jones, Daisuke Kohno, Kevin W. Williams, Charlotte E. Lee, Michelle J. Choi, Jason G. Anderson, Lora K. Heisler, Jeffrey M. Zigman, Bradford B. Lowell, and Joel K. Elmquist

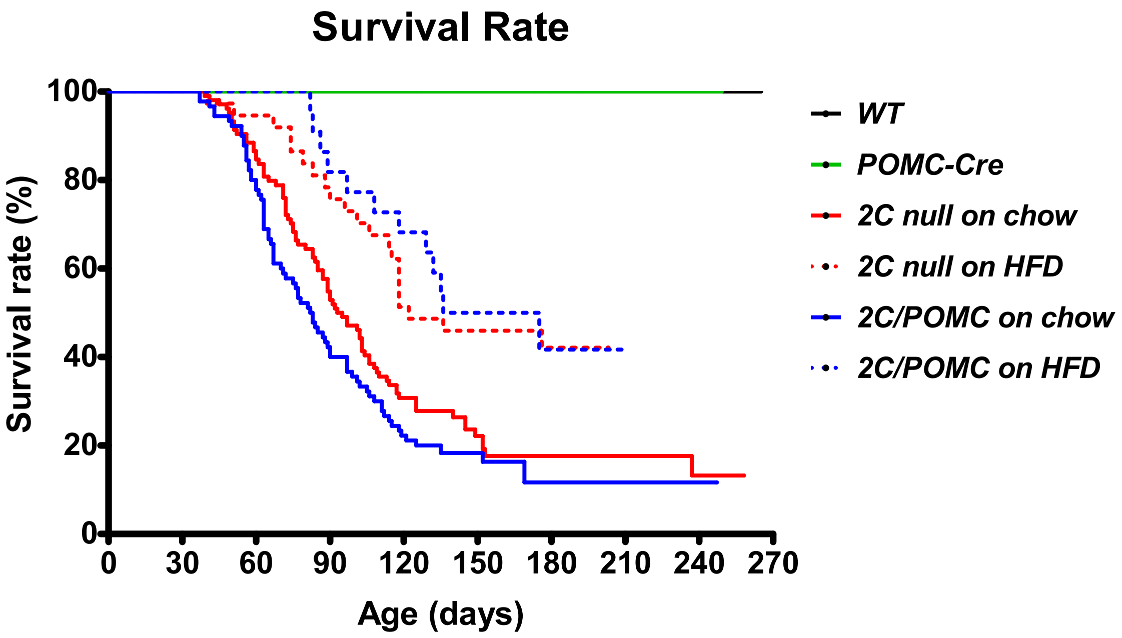

Supplemental figure 1. Selective re-expression of 5-HT$_{2C}$Rs in POMC neurons fails to rescue decreased survival rate. *2C null* mice on chow (n = 104) showed significantly decreased survival rate than *WT* (n = 97) and *POMC-Cre* (n = 81) mice; survival rate of *2C null* mice on HFD (n = 37) was significantly higher than *2C null* mice on chow. *2C/POMC* mice, either on chow (n = 90) or on HFD (n = 22), showed comparable survival rate as their *2C null* littermates.
